# Editorial: Emerging issues in public health

**DOI:** 10.3389/fpubh.2023.1260924

**Published:** 2023-09-05

**Authors:** Paolo Vineis

**Affiliations:** Department of Epidemiology and Biostatistics, Imperial College, London, United Kingdom

**Keywords:** finance, equity, global health, aging, occupational risks, minorities

## Introduction

Why this Research Topic of Frontiers in Public Health on “*Emerging issues in public health*?” Public health has become popular during the COVID-19 pandemic, and a large number of people have learnt the terminology and concepts of the scientific discipline that supports it, epidemiology. However, very few appreciated the involvement of epidemiology and public health in almost every aspect of medicine and beyond medicine, encompassing for example the quality of all medical treatments; the evidence underlying policies to tackle social inequalities; the health impacts of climate changes; and many others.

In this Research Topic we present a few examples of applications of the epidemiological and public health methods to address emerging societal issues, that require bright policy solutions. Needless to say, the relationship between science and policy is not straightforward. It would be naïve to think that scientists provide evidence and then this is translated into policy. Policy-making is the integration of science and values and requires trade-offs and people's participation. However, confused and polarized communication of evidence—largely due to the role played by the new media (1)—makes the relationship between science, civil society and politics complex and frustrating. The fragmentary and speedy nature of the world of communication is paralleled by the slow pace of the world of politics. In the meantime, although there is a long and healthy tradition of distancing science from direct political engagement (that is healthy in the sense that the scientist is expected to investigate nature without being swayed by his or her ideological preferences), climate change challenges a clear-cut separation between science and advocacy. Without the youth movements, for example, that of scientists would be “vox clamantis in deserto” (a voice calling in the desert) in the case of the environmental crisis.

## The world of business

Among the challenges posed by the environmental crisis, namely to public health, there is the problem of relationships with the business world. I will argue that it is legitimate and useful that public health looks to the world of business and finance. Indeed, ignoring the corporate world and finance in particular runs the risk of being a serious scotoma among those concerned with climate and health. Businesses produce much of the income but they are also responsible for much of the pollution and greenhouse gases. They are responsible for most of the lobbying to politicians. On the finance side, they are involved in the “great acceleration” that characterizes contemporary capitalism, i.e., a heightened rapidity in the movement of money, goods and people (with all the environmentally destructive implications), and substantial volatility and instability in the economic system. Attempts have been made to curb these inherent trends, for example through the ESG rating of investment funds. ESG stands for “Environment, Social and Governance;” i.e., it is an acronym that induces companies to be responsible to the environment and society and to deal with governance, that is, the downstream consequences (the diseconomies) of their activities. But ESG ratings are completely unreliable even according to the most prudent sources: the Economist devoted a Research Topic (2) (significantly titled “there is a need for cleanup”) to them, in which all the failures of ESG are mercilessly listed, and are basically summarized in the term “greenwashing.” ESG ratings have been a huge success in the financial market: between 2015 and 2019, when the assets of ESG-certified companies increased by 500%. But ESGs have enormous limitations: it is not clear what information they are based on; ratings are assigned by a multiplicity of firms using different criteria and methods that are not comparable; they refer to the present and not to the future (i.e., not to the investments companies make to improve their environmental and social performance).

If we want to impact climate change, we cannot ignore these phenomena. The effectiveness of ESG comes from the modest (to say the least) ethos of the finance world, but also from the great speed and volatility of capital investments. That is why new ways of evaluating investment funds are being explored, such as the Long-term Stock Exchange, the development of mechanisms that measure success not in terms of days, hours, or minutes, but in terms of years; and that are based on long-term oversight. It is unclear whether capitalism will be able to amend itself, but in the absence of a credible alternative, it is incumbent on us to pay attention to those forces that seek to reform it from within, such as the B-Corps, the companies that have clauses in their bylaws that stipulate that they will not put profit above concern for the environment and people's health. And more generally to that vast world of businesses, large and small, that have begun to realize that veering toward the green transition may be the best investment for the future. These are the topics addressed in the Perspective on “*The need for new metrics in the Anthropocene era*” by Vineis and Mangone, in the present Research Topic.

## The various facets of health inequalities

Coming immediately after climate change in importance, and concurrent with it, is social inequality. In fact, the environmental crisis and inequalities go hand in hand, and it is broadly agreed that tackling the first requires strong policies against the second. This Research Topic has a number of contributions that address social inequalities in health in a broad sense. First, we can read a contribution on young workers, a kind of neglected population. This is an extremely important field of research: many young workers today are characterized by huge instability and uncertainty, and are exposed to risk factors among which stress, disruption of circadian rhythms and lack of sleep are common. However, workers in the gig economy are very difficult to investigate because of the absence of regular and systematic registries of workers like in traditional industries, and for the general instability of this workforce. In addition, they are young and may not show yet signs of overt disease for many years. According to the *Perspective: young workers at higher risk for carcinogen exposures*, workers under the age of 25 are usually considered a vulnerable working population, primarily due to their risk of injury. In their study Sweet et al. investigated if young workers may also be at an increased risk for occupational exposure to carcinogens. Based on CAREX Canada data, they show that young workers in construction, outdoor occupations, and farming are key groups that warrant further investigation. This conclusion is reached because of the large number of young workers employed in those sectors, the high number of possible carcinogen exposures, and the potential for high risk behavioral patterns in these types of jobs. It is clearly necessary to improve the occupational health and safety measures for this vulnerable population. In addition, innovative ways to address occupational risks in the gig economy need to be developed, as has been suggested before in this journal (3).

Concerning biomarkers, like those proposed in Freni-Sterrantino and Salerno (3), one of the needs is not only to develop markers able to predict diseases long before their onset, but also to consider a life-course perspective. This is tackled in another contribution to this Research Topic, by Lin and Appleton (*Developmental origins of pregnancy-related morbidity and mortality in black U.S. women*), related to inequalities involving gender and race. In the US, Black women have a disproportionately high risk of pregnancy-related morbidity and mortality. Research on the determinants of such disparities has focused predominantly on risk factors occurring during pregnancy, while a set of studies has investigated the developmental origins of health and disease (DOHaD) model *but only in a limited way in Black people*. This stream of research seems to indicate that the origins of adult cardiometabolic health can be traced back to stressors occurring during the intrauterine and early life periods. Lin and Appleton argue that the DOHaD model represents a theoretical framework from which to conceptualize factors that drive racial disparities. This is a very important conclusion that requires up-to-date research including novel biomarkers (4) and more attention to minorities.

Still related to inequalities, in this case associated with exposure to SARS-CoV-2, is the issue of education and its disruption by the pandemic, particularly among the low socio-economic groups. Stock et al. argue in their review of the literature that closures of institutions for face-to-face teaching over a long period of time have had significant consequences on the psychosocial health and wellbeing of students in many countries. The perspective (*COVID-19 related disruption in higher education students' health and wellbeing: implications for university action*) advocates for health promotion and support services, programmes, structures and policies together with the wider policy-oriented approach of a Health Promoting University.

Elliott et al. tackle another issue that has become urgent in the last decades in all high-income countries and the middle class of low- and middle -income countries, the quality of diet and food literacy (*Food promotion and children's health: considering best practices for teaching and evaluating media literacy on food marketing*).

Food marketing to children tends to promote foods of poor nutritional quality and influences children's food preferences and habits. It is therefore a factor in childhood obesity (see for example the results of the EU-funded network STOP: https://www.stopchildobesity.eu/). Children and their parents urgently need critical literacy skills that increase their understanding of food marketing. This paper shows the outcomes of a stakeholder meeting on best practices in literacy and food marketing to children, suggesting useful criteria. Needless to say, once again this is a topic strictly related to social inequalities: for example, a study in Brazil has found large differences in the intake of ultraprocessed food (UPF) by socio-economic position (5). UPF in turn has been found to be associated to childhood obesity (6), and its consumption is clearly affected by marketing strategies.

Finally, Smith and Ory address still another topic that is affected by large social inequalities, falls in the older population (*Multi-directional nature of falls among older adults: a rationale for prevention and management*). Their aim is to to challenge the way we think about falls, that should be viewed as a *multi-directional occurrence*. A fall may be the result of a set of personal or environmental circumstances, a sign or symptom of an underlying health issue, or a cause of subsequent health consequences. This leads to the claim that we must holistically assess the person and determine the role of the fall within the context. Among many other determinants, loneliness is a strongly socially patterned situation and a strong risk factor for falls itself, acting as a multiplier of other risk factors (depression, cognitive impairment, sedentary behavior, and substance use). While clinicians have implemented strategies to refer at-risk older adults to fall prevention programs, it is also necessary to consider how falls interact with other health issues (e.g., malnutrition, physical inactivity, cognitive impairment, social disconnectedness, polypharmacy, and the built environment). Additional multi-level, multi-sectorial solutions are needed to prevent and manage falls, including research, healthcare practice, community programming, and policy to ensure “practical, effective, replicable, scalable, and sustainable” solutions.

This editorial does not exhaust all the richness of the papers in the Research Topic, and just highlights some common aspects, the main of which is inequalities and vulnerable subgroups in the population. The conclusion of the papers in the Research Topic is not the usual one that “more research is needed,” but, consistently with the spirit of this journal, “more evidence needs to be transferred into public health practice.” Thoughtful and novel solutions are proposed and should become priorities in the prevention agenda of health and social services addressing young workers, the black population and other racial minorities, women, the older population and children (particularly the deprived ones). Special considerations are needed for climate change and environmental degradation, that require quick action taking into account several degrees of vulnerability in the population.

## Author contributions

PV: Writing—original draft.

## References

[1] Quercus. Max Fisher: The Chaos Machine. London: Quercus (2022).

[2] The Economist. In Need of a Clean-Up. London: The Economist (2022).

[3] Freni-Sterrantino A Salerno V. A plea for the need to investigate the health effects of gig-economy. Front Public Health. (2021) 9:638767. 10.3389/fpubh.2021.638767 33634072 PMC7900166

[4] Demetriou CA van Veldhoven K Relton C Stringhini S Kyriacou K Vineis P. Biological embedding of early-life exposures and disease risk in humans: a role for DNA methylation. Eur J Clin Invest. (2015) 45:303–32. 10.1111/eci.12406 25645488

[5] Handakas E Chang K Khandpur N Vamos EP Millett C Sassi F. Metabolic profiles of ultra-processed food consumption and their role in obesity risk in British children. Clin Nutr. (2022) 41:2537–48. 10.1016/j.clnu.2022.09.002 36223715

[6] Viola PCAF Ribeiro SAV Carvalho RRS Andreoli CS Novaes JF Priore SE. Socioeconomic status, screen time, and time spent at school, and children's food consumption. Cien Saude Colet. (2023) 28:257–67. 10.1590/1413-81232023281.05772022 36629570

